# An *in silico *study of the molecular basis of B-RAF activation and conformational stability

**DOI:** 10.1186/1472-6807-9-47

**Published:** 2009-07-22

**Authors:** Filip F Fratev, Svava Ósk Jónsdóttir

**Affiliations:** 1 Center for Biological Sequence Analysis, Department of Systems Biology, Technical University of Denmark, Kemitorvet, Building 208, DK-2800 Kongens Lyngby, Denmark

## Abstract

**Background:**

B-RAF kinase plays an important role both in tumour induction and maintenance in several cancers and it is an attractive new drug target. However, the structural basis of the B-RAF activation is still not well understood.

**Results:**

In this study we suggest a novel molecular basis of B-RAF activation based on molecular dynamics (MD) simulations of B-RAF^WT ^and the B-RAF^V600E^, B-RAF^K601E ^and B-RAF^D594V ^mutants. A strong hydrogen bond network was identified in B-RAF^WT ^in which the interactions between Lys601 and the well known catalytic residues Lys483, Glu501 and Asp594 play an important role. It was found that several mutations, which directly or indirectly destabilized the interactions between these residues within this network, contributed to the changes in B-RAF activity.

**Conclusion:**

Our results showed that the above mechanisms lead to the disruption of the electrostatic interactions between the A-loop and the αC-helix in the activating mutants, which presumably contribute to the flipping of the activation segment to an active form. Conversely, in the B-RAF^D594V ^mutant that has impaired kinase activity, and in B-RAF^WT ^these interactions were strong and stabilized the kinase inactive form.

## Background

The identification and characterisation of functional targets that play an essential role in tumour growth and survival, is of imperative importance to the success of novel cancer therapies. B-RAF, a member of the RAF kinase family, is an important new therapeutic target for a group of human cancers, as it constitutes activation of the RAF-MEK-ERK pathway common to numerous cancers [1]. Recently, large-scale genomic screens have detected mutations in B-RAF in about 70% of malignant melanomas and at a lower frequency in colorectal and ovarian cancers [2]. Other cancer types have been found to harbour B-RAF mutations. Over 40 mutations have already been identified [3,4]. Some of these mutants, B-RAF^E586K^, B-RAF^V600E^, B-RAF^V600D^, B-RAF^V600K^, B-RAF^V600R ^and B-RAF^K601E^, have a much higher kinase activity *in vitro *than the basal wild type B-RAF (B-RAF^WT) ^activity and were classified as strongly activating [4]. Mutations at nine amino acid positions that are less activating, but exhibit activity above the basal, belong to an intermediate activity group [4]. Although most B-RAF mutants display elevated kinase activity compared to the B-RAF^WT^, four cancer-derived mutants have reduced kinase activity: B-RAF^G466E^, B-RAF^G466V^, B-RAF^G596R ^and B-RAF^D594V ^[3,4].

Most of the mutations of B-RAF are clustered in two different regions within the binding pocket, one is the glycine-rich P-loop (P-loop) of the N lobe and the other one is the activation segment (named A-loop here). A substitution of Val by Glu at residue 600 in the A-loop, adjacent to the conserved DFG motif, accounts for 90% of B-RAF mutations in human cancers. B-RAF^V600E ^has a 500-fold higher kinase activity, relative to the basal activity of B-RAF^WT^, providing cancer cells with both proliferation and survival signals and allowing them to grow as tumours in model systems [4].

The activation of B-RAF^WT ^requires phosphorylation of the Thr599 and/or Ser602 residues within the A-loop [5]. It has been suggested that the V600 mutations mimic the phosphorylation step [2], and has also been stated that most of the B-RAF mutants can activate MEK directly and thereby stimulate ERK [3,4]. However, the mutated and activated B-RAF^V600E ^binds to a Hsp90-cdc37 complex, which is required for its stability and function [6]. Three of the impaired kinase activity mutations (B-RAF^G466E^, B-RAF^G466V ^and B-RAF^G596R^) are capable of inducing ERK phosphorylation through heterodimerization with C-RAF. The fourth mutant (B-RAF^D594V^) acts like a kinase-dead mutant and cannot bind to C-RAF. Its role in tumorigenesis remains to be elucidated [3]. It is not surprising that B-RAF^D594V ^is a loss-of-function mutant, because Asp594 is a key catalytic residue for many kinases [7]. The impact of the D594V mutation on the B-RAF conformation, and in particular on the coordination and interaction with ATP binding residues such as Lys483, Glu501 and Asp594 is yet unclear.

Crystal structures of the inactive B-RAF^WT ^and B-RAF^V600E ^kinase binding domains in a complex with the BAY-439006 (Sorafenib) inhibitor have been solved [4]. It has been suggested that the molecular interactions between the P-loop and the A-loop in B-RAF^WT ^stabilize the inactive form, and that the substrate recognition leads to conformational changes that set the catalytic cleft free and allows the enzyme to achieve a full active state [4,8]. In particular, interactions between Phe468 in the P-loop and Val600 in the A-loop were identified and based on this observation it has been predicted that the mutations of Val600 to a larger and more charged residues will lead to conformational change within the A-loop, flipping it to the active position. Such structural model could also explain the strong increase in the kinase activity caused by the G469A mutation in the P-loop and intermediate increase in activity for a group of other P-loop mutants. However, the molecular basis of the strong B-RAF activation by the K601E mutation remains unclear, and it is also difficult to explain the strong kinase activity by the B-RAF^E586K ^mutant, and the intermediate activation by the N581S, F595L, L597V, L597R and T599I mutations within in frame of the present literature model, because none of these residues are in a close contact with the P-loop. Moreover, the position of Phe468 in a newly obtained crystal structures of the inactive B-RAF^WT ^[9] differs significantly from the corresponding position in previously solved crystal structures. The new data showed that in a presence of a ligand smaller than Sorafenib or with no inhibitor present in the binding site, Phe468 has an orientation toward the αC-helix and is unlikely to interact with Val600. The results from MD simulations of B-RAF^WT^, B-RAF^V600E ^and B-RAF^V599Ins ^showed that the electrostatic interactions between the A-loop, the P-loop and the αC-helix are an important factor for the conformational stability of the activation segment in B-RAF^WT ^and in the mutations causing disruption of these interactions, but the particular contribution of the αC-helix to these processes was not specified [10]. Further, the molecular bases for the ligand inhibition, selectivity and conformation stability in inactive B-RAF^WT ^and B-RAF^V600E ^has been studied, indicating that the αC-helix – A-loop interactions play a crucial role in B-RAF activation. In this study the Lys601 residue in the A-loop and the Glu501 residue in αC-helix were identified to provide the strongest electrostatic component to the overall interactions within the protein-ligand complex [11].

Recently, crystal structures of the active form of B-RAF (B-RAF-A), i.e. the kinase with the A-loop moved to its active position, were obtained [9,12]. The conformational change of the A-loop is a major structural feature that occurs during the kinase activation [3,4,12]. Due to its conformational flexibility, some of the residues in the A-loop were not resolved in all available crystal structures of the active as well as inactive B-RAF forms.

In the present paper we suggest a molecular basis for several of the experimentally observed B-RAF kinase activity deviations induced by the mutations. We study the direct and indirect changes caused by the mutants on a strong hydrogen bond network identified in the B-RAF wild type, and put forward a hypothesis on how the observed structural changes contribute to the experimentally measured kinase activity deviations.

## Methods

### Preparation of protein structures

Firstly, the structures of B-RAF^WT ^and B-RAF^V600E ^were prepared as described in a previous publication [11]. By retrieving the atomic coordinates of available crystal structures for B-RAF^WT ^and B-RAF^V600E ^from the protein data bank [13] (pdb codes: 1uwh and 1uwj) and removing the inhibitor Sorafenib from the protein while keeping the crystal waters, initial structures for these B-RAF forms were generated. The loop building routine of Modeller [14] was used to create models for the regions not solved into the crystal structures, which are residues 601–612 for B-RAF^WT ^and 603–614 for B-RAF^V600E^, as describe previously [11].

The mutants B-RAF^K601E ^and B-RAF^D594V ^were constructed using the crystal structure of B-RAF^WT ^in each case by substituting the corresponding residue with the mutation. Then the A-loop region was modelled and refined using the same procedure as was used for the two other B-RAF forms.

### Molecular dynamics simulations

For each of the B-RAF kinases studied, a 15 ns MD simulation was done. The structures of the different B-RAF forms were prepared with the Amber 9 software [15] and all the MD simulations were done with NAMD v.2.6 [16]. All simulations were carried out at neutral pH. The Lys and Arg residues are positively charged, and the Asp and Glu residues are negatively charged. The default His protonated state in Amber 9 was adopted. Each of the simulated systems was placed in the centre of a cubic simulation box filled with water molecules. The SPC/E water model [17] was used for the waters and the buffering distance was set to 10 Å. Counter ions were added to maintain the electroneutrality of the complexes. The Amber ff03 force field was employed for the simulations [18]. All systems were energy minimized in two steps. Firstly only the water molecules and ions were minimized for 2000 steps while keeping the protein structure fixed. Secondly, a 8000 step minimization with the conjugate gradient method to convergence criterion of 0.5 kcal mol^-1 ^Å^-1 ^was performed on the whole system (waters, ions and protein). The simulated systems (the different B-RAF forms) were then gradually heated from 0 to 300 K for 50 ps (NTV), equilibrated for 200 ps, and finally production runs of 15 ns (NTP) were performed. Langevin dynamics was used to keep the temperature constant throughout the simulation time, setting the Langevin damping coefficient to 5 ps^-1^. The hybrid Nosé-Hoover Langevin piston method with a decay period of 200 fs and a damping time-scale of 50 fs was used to keep the pressure constant at 1 atm. A non-bonded cut-off of 12.0 Å and the switching distances were imposed to10 Å and 14 Å respectively. The SHAKE algorithm was used for all bonds involving hydrogen atoms [19]. A 2 fs integration time step was used, and the particle-mesh Ewald (PME) method [20] was applied to model the long-range electrostatic interactions. Data were collected every 500 steps, i.e., every 1 ps. The average distances and corresponding standard deviations (SD) were calculated with the VEGA software [21]. The interactions energies between selected protein substructures and residues were calculated with NAMD. Calculation of the root-mean-square deviations (RMSD), the root-mean-square fluctuations (RMSF) and the corresponding SD values and visualisations of the protein structures were done with the VMD package and its Tcl routine [22].

## Results and discussions

### Overall molecular dynamics analysis

In order to establish a link between the structural changes that occur within the binding pocket of B-RAF and the experimentally observed activity deviations, a detailed analysis of the MD simulation results was performed. Thus structural analysis of the B-RAF wild type and the three mutants is used as bases for a hypothesis on the effect the various mutation inflict on the B-RAF activity on ERK protein.

**Figure 1:**
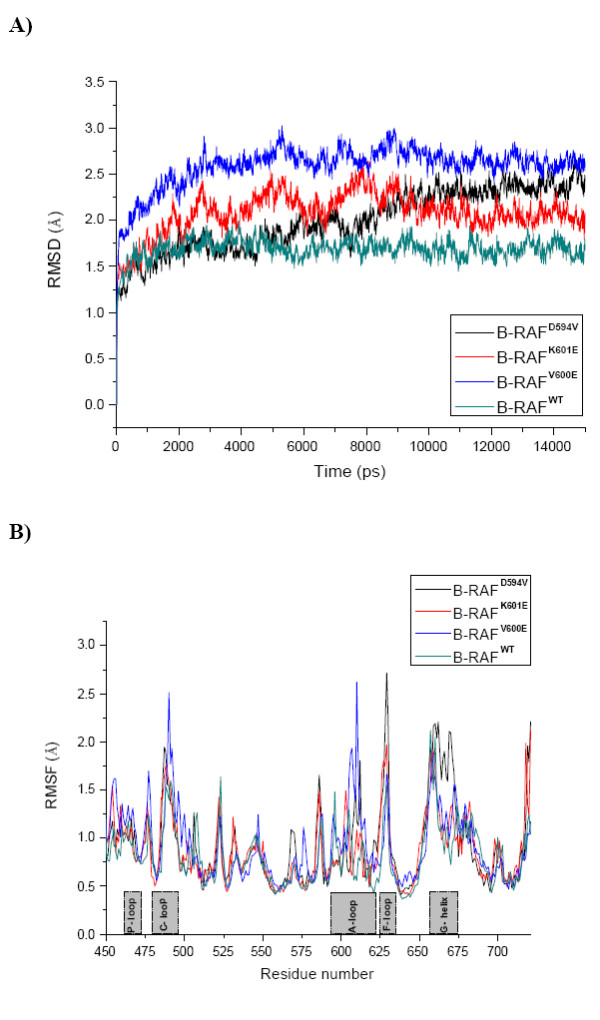
**Overall molecular dynamics analysis**. **A) **The RMSD of the proteins backbone atoms (C, Cα and N atoms) for the different kinase forms, B-RAF^WT^, B-RAF^V600E^, B-RAF^K601E ^and B-RAF^D594V^. **B) **The RMSF of the proteins Cα atom for each residue of the different kinase forms, B-RAF^WT^, B-RAF^V600E^, B-RAF^K601E ^and B-RAF^D594V^.

**Figure 2:**
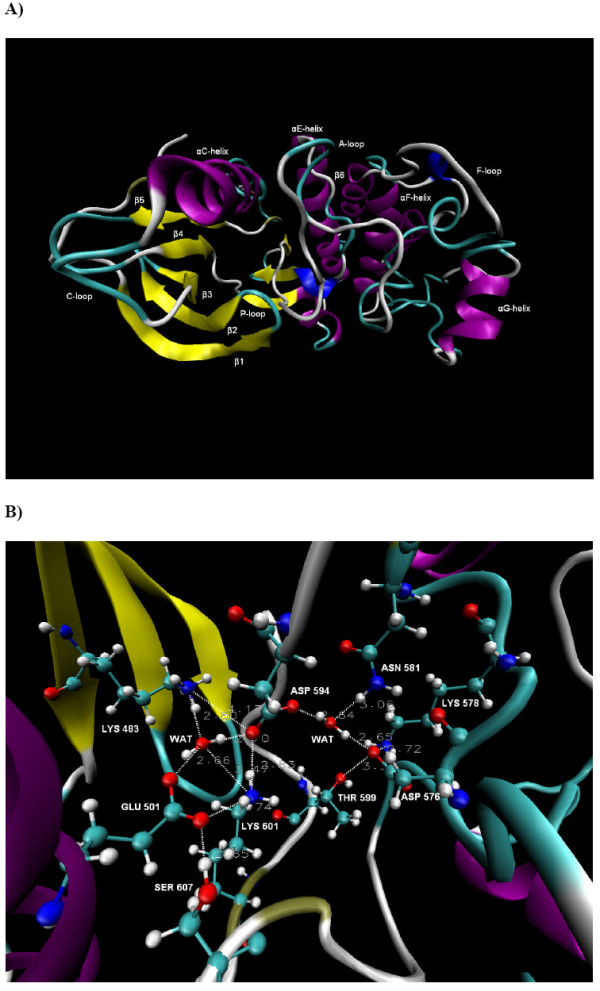
**B-RAF^WT^**. **A) **The conformation of B-RAF^WT ^after 15 ns of simulation time. The different structural elements in the protein are coloured according to the structure method in VMD and the names of the sub-structures are shown. The A-loop contains 30 residues and is in the space between the P-loop, the αC-helix, the β6-strand, the F-loop and the αG-helix (see text for details). **B) **A close look at the conformation of the major catalytic residues and the identified hydrogen bond network in the unbounded B-RAF^WT ^after 15 ns of simulation time (The position of Gly596 is shown in Figure 7). The dotted lines and the adjacent numerical values represent the H-bonds and their distances.

Figures 1A and 1B present the RMSD of the backbone proteins atoms during the 15 ns of simulation and the RMSF of the Cα atom in each residue, respectively. The wild type protein reached a reasonable equilibration after 1.5 ns whereas for the mutants it took 3.5 ns until equilibrium was reached. A good RMSD convergence for all the three mutants was reached after 9 ns. These data are consistent with recently obtained MD results for the structurally similar Abl kinase [23], suggesting that at least a few nanoseconds of equilibration are required to study these systems. It was observed that both the active mutants, B-RAF^V600E ^and B-RAF^K601E^, have a similar evolution of the RMSD curve, whereas the curve for the inactive mutant, B-RAF^D594V^, evolves differently. However, the observed similarity between the two curves could be an artefact of the A-loop missing portion model. Each form of the B-RAF reaches stable equilibrium conformation, which are used for the analysis presented in the paper. The analysis of the RMSF graph showed that there were four major flexible protein elements present in all the studied proteins. These are the loop that links the β3 strands and the αC helix (named C-loop here), the loop between the A-loop and the αF-helix (named F-loop here), the A-loop and the αG-helix (see Figures 1B and 2A). The fluctuations of the residues were higher in magnitude in the mutants than for the wild type. The A-loop residues flexibility was high and varied in the individual kinases, but was most strongly expressed in the V600E mutant.

In order to study the contribution of the protein structural elements to the A-loop kinase conformational stability, the van der Waals and electrostatic interactions between the A-loop and the surrounding protein sub-structures were calculated. The interactions between the A-loop (residues 593–623) and the closest five neighbouring sub-structures, the P-loop (residues 462–471), the αC-helix (residues 492–506), the β6 strand (residues 571–578), the F-loop (residues 625–634) and the αG-helix (residues 660–672), were analysed, see Tables 1A and 1B. An investigation of the energy contributions of the individual protein residues to these processes was performed too. The data were extracted for the period 10–15 ns of simulation time when a good convergence was reached for all the four B-RAF forms. A similar approach was recently used for B-RAF^WT^, B-RAF^V600E ^and B-RAF^V600Ins^, but the particular interactions between the A-loop, the αC-helix and the P-loop, as well as the contribution of the other sub-structures, were not specified [10].

**Table 1:** An overview of the calculated interaction energies (kcal/mol) between key sub-structures and residues in A) B-RAF^WT ^and B-RAF^V600E ^and in B) B-RAF^K601E ^and B-RAF^D594V^.

**A)**	**B-RAF^WT^**			**B-RAF^V600E^**		
Substructure	vdW	El	Total	vdW	El	Total

A-loop^a^	-100.9 ± 6.9	-200.1 ± 18.6	-301.0 ± 16.5	-99.1 ± 5.6	-153.6 ± 24.4	-252.7 ± 24.5
A-loop-αC-helix^b^	-20.2 ± 1.9	-31.4 ± 5.9	-51.6 ± 5.4	-27.0 ± 3.2	-5.8 ± 10.6	-32.8 ± 9.9
A-loop-P-loop^c^	-12.7 ± 2.2	-18.2 ± 5.5	-30.9 ± 5.1	-22.6 ± 2.0	-11.9 ± 2.8	-34.5 ± 3.2
A-loop-β6-strand^d^	-12.4 ± 2.7	-31.6 ± 8.6	-44.0 ± 8.4	-14.2 ± 2.1	-44.5 ± 9.3	-58.7 ± 8.9
A-loop-F-loop^e^	-18.5 ± 1.8	-3.4 ± 4.4	-21.9 ± 4.1	-15.3 ± 2.7	-20.3 ± 23.5	-35.6 ± 23.4
A-loop-αG-helix^f^	-3.6 ± 1.3	-33.6 ± 7.4	-37.2 ± 7.3	-0.7 ± 0.3	-6.4 ± 2.5	-7.1 ± 2.6
Lys601^g^	-14.9 ± 2.3	-150.3 ± 15.1	-165.2 ± 14.8	-15.8 ± 2.3	-137.7 ± 12.7	-153.5 ± 12.1
Lys601-αC-helix^h^	-0.7 ± 1.5	-73.3 ± 9.9	-74.0 ± 9.2	-3.2 ± 0.5	-22.8 ± 3.1	-26.0 ± 3.1
Lys483-Asp594^i^	-0.1 ± 0.9	-69.0 ± 9.5	-69.1 ± 9.0	-0.3 ± 0.1	-39.4 ± 4.4	-39.7 ± 4.5
Lys601-Asp594^j^	-0.2 ± 0.6	-58.2 ± 4.2	-58.4 ± 4.0	-1.5 ± 3.9	-61.5 ± 4.5	-63.0 ± 3.4
(K601-D594)-^k^ (E501-K483)	-0.9 ± 1.7	-70.8 ± 7.5	-71.7 ± 6.7	-4.0 ± 0.5	0.2 ± 3.5	-3.8 ± 3.5
(K601-D594)-^l^ (D576-N581)	-1.4 ± 0.4	2.3 ± 3.3	0.9 ± 3.1	-2.1 ± 1.5	-40.3 ± 8.7	-42.4 ± 8.1
**B)**	**B-RAF^K601E^**			**B-RAF^D594V^**		

Substructure	vdW	El	Total	vdW	El	Total

A-loop^a^	-117.7 ± 5.8	-261.5 ± 26.4	-379.2 ± 26.6	-105.6 ± 5.3	-108.8 ± 12.2	-214.4 ± 12.8
A-loop-αC-helix^b^	-34.2 ± 2.9	-9.2 ± 12.2	-43.4 ± 12.2	-32.7 ± 2.7	-27.7 ± 7.9	-60.4 ± 7.6
A-loop-P-loop^c^	-16.2 ± 1.8	-18.4 ± 3.8	-34.6 ± 4.4	-20.0 ± 1.7	-1.9 ± 2.4	-21.9 ± 2.9
A-loop-β6-strand^d^	-17.9 ± 2.2	-26.9 ± 7.1	-44.8 ± 7.2	-18.5 ± 2.4	-35.1 ± 7.7	-53.6 ± 7.0
A-loop-F-loop^e^	-2.6 ± 0.7	-0.2 ± 1.6	-2.8 ± 1.8	-13.9 ± 2.6	1.1 ± 3.7	-12.8 ± 5.0
A-loop-αG-helix^f^	-2.3 ± 1.2	-56.5 ± 8.3	-58.8 ± 8.4	-0.07 ± 0.1	0.12 ± 1.7	0.05 ± 1.7
Lys601^g^	-12.1 ± 2.6	-141.6 ± 7.2	-153.7 ± 6.8	-20.3 ± 2.2	-102.9 ± 12.8	-123.2 ± 12.7
Lys601-αC-helix^h^	-5.6 ± 1.4	-10.8 ± 4.5	-16.4 ± 4.1	-5.8 ± 0.8	-29.6 ± 3.0	-35.4 ± 2.9
Lys483-Asp594^i^	-0.4 ± 0.6	-52.8 ± 7.7	-53.2 ± 7.5	-0.02 ± 0.0	1.06 ± 0.3	1.04 ± 0.3
Lys601-Asp594^j^	-0.3 ± 0.1	36.4 ± 3.4	36.1 ± 3.3	-0.8 ± 0.7	-6.6 ± 1.9	-7.4 ± 1.7
(K601-D594)-^k^ (E501-K483)	-2.0 ± 1.4	-48.9 ± 5.7	-50.9 ± 5.5	-4.0 ± 0.6	-9.1 ± 2.1	-13.1 ± 2.0
(K601-D594)-^l^ (D576-N581)	-2.9 ± 0.5	60.9 ± 4.2	58.0 ± 4.0	-2.6 ± 1.5	-79.0 ± 6.8	-81.6 ± 6.7

^a^The interactions between the A-loop (residues 593–623) and the remaining protein. ^b, c, d, e, f^The interactions between the A-loop and the αC-helix (residues 492–506), the P-loop (residues 462–471), the β6 strand (residues 571–578), the F-loop (residues 625–634) and the αG-helix (residues 660–672), respectively. ^g, h ^The interactions between Lys601 and the remaining protein, and Lys601 and the αC-helix, respectively. ^i ^The interactions between Lys483 and Asp594. ^j^The interactions between Asp594 and Lys601.^k, l ^The interactions between the Asp594-Lys601 and Lys483-Glu501 ion pairs and the Asp594-Lys601 and Asp576-Asn581 pairs, respectively.

The van der Waals and the electrostatic contributions to the total interactions energies averaged for the period 10–15 ns of simulation time, along with the measured standard deviations, are listed.

Furthermore, the evolution of the electrostatic interaction energy between key residues throughout the whole simulation time was plotted, and used to examine the structural changes occurring within the binding pocket of B-RAF during the simulation. As part of the A-loop was not resolved in the available crystal structures, the contribution of the modelled A-loop portion to the overall structural changes of the different B-RAF mutations had to be evaluated. Thus the A-loop region of the mutants was further divided into two portions, A-loop1 (residues 593–602 resolved in the crystal structure) and A-loop2 (residues 603–614 not solved in the crystal structure), and interaction energies between these two portions and other keys residues were studied separately.

This analysis was focused on structural changes occurring within the ATP binding site. The electrostatic and van der Waals interaction energies between key residues and protein sub-structures were calculated, but energy deviations to due to solvation of the different protein sub-structures were not calculated specifically. However, the MD simulations were performed in the presence of water, and thus we consider the resulting protein structures to be governed by all relevant interaction within the systems studied, including hydrophobic and other solvation effects, and electrostatic, van der Waals and intra-molecular forces. It was seen that forming and breaking of H-bonds led to very significant structural changes, and such interactions are reasonably well described by the electrostatic and the van der Waals interactions. These energy terms were thus considered to provide sufficient information for this analysis. The effects due to other interactions like the hydrophobic ones could thus only be discussed in qualitatively terms

### Molecular dynamics of B-RAF^WT^

Figures 2A and 2B show the B-RAF^WT ^conformation and the position of the major catalytic residues after 15 ns of MD simulation. A two part inter-linked water-mediated hydrogen bond network (HBN) was identified and remained stable during the simulation time. The first HBN was formed between Lys483, Glu501, Asp594, Gly596, Lys601, Ser607 and water molecules (HBN1). Asp594 and Lys601 were also joined in a second water-mediated hydrogen bond bridge with Lys578, Asn581, Asp576 and Thr599 (HBN2). Thus a unique and inter-linked HBN situated in the space between the αC-helix, β3 strand, A-loop and β6 strand, was identified. These results thus confirm the previous results from a 10 ns MD simulation, where a similar HBN was identified [11].

There are five protein elements that interact with the A-loop. These are the F-loop, P-loop, αG-helix, αC-helix and β6 strand, with both the P-loop and the αC-helix in close contact with the A-loop residues that participate in the above mentioned HBN. The total interactions between the A-loop and the αC-helix constituted 17% of total A-loop-protein interactions, whereas the P-loop-A-loop interactions contributed with 10% (see Table 1A).

The H-bond between Glu501 and Lys601 was observed during 94% of the simulation time with an average distance of 2.91 Å ± 0.38. The average distances of Lys483-Asp594 and Lys601-Asp594 were 3.92 Å ± 0.87 and 3.43 Å ± 0.80, respectively. Direct hydrogen bond between these residue pairs were observed during 75% and 65% of the simulation time, respectively. Thus according to the simulation results, Lys601 and the three catalytic residues, Lys483, Glu501 and Asp594, interact strongly via tight net of hydrogen bonds.

The closer distance between Lys483 and Asp594 compared with those seen in the crystal structures (6.02 Å [4]) is due to the lysine conformational change and an orientation toward Asp594. Thus, it appears that the interactions between these catalytic residues in the unbounded B-RAF^WT ^are stronger than the corresponding interactions in the bound states, where the inhibitors most likely affect their conformation [4,9,11,12].

The average electrostatic interaction energy between Lys483 and Asp594 was -69.0 ± 9.5 kcal/mol, which is 35% of the total A-loop-protein electrostatic interactions (see Table 1A). The corresponding value for the Lys601-Glu501 ion pair was -93.8 ± 3.5 kcal/mol, which equals to 47% of the electrostatic A-loop-protein interactions. Moreover, the Lys601-Glu501 ion pair contributes with 31% of the total A-loop-protein interaction energy (van der Waals plus electrostatic). The average electrostatic interaction energy between the Lys601-Asp594 and the Lys483-Glu501 ion pairs was -70.8 ± 7.5 kcal/mol. The above results reveal strong interactions between the αC-helix residue Glu501 and the A-loop residues Asp594 and Lys601, and thus it is fair to suggest that the interactions between these two protein substructures presumably have significant effect on the conformational stability of B-RAF^WT^. The strong interactions between Lys601 and the known catalytic and ATP coordinating residues Asp594, Glu501 and Lys483 observed previously [11] was thus seen to be maintained throughout the 15 ns MD simulation. The results indicate that Lys601 has an important role in the B-RAF^WT ^function and stability, a finding which is further supported by the experimentally observed strong kinase activation in the presence of the K601E mutation. According to these results, one can speculate that Lys601 plays an important role in the inactive B-RAF^WT ^phosphorylation, because it interacts strongly with the ATP coordinating residues and could possibly act as a regulator of these processes. Moreover, the interaction between Asp594 and Lys601 was detected in the crystal structure of the V600E mutant, indicating that our results were not affected by the loop modelling of a part of the A-loop [4].

The Lys601 and Glu501 residues were previously identified to be of key importance to the protein-ligand interactions of B-RAF^WT ^and B-RAF^V600E ^in complexes with a series of inhibitors. This study was based on a detailed analysis of MD simulation results, as well as statistical analysis showing good correlation between the biological activity and key energy contributions, thus also providing experimental bases for our hypothesis [11].

We observed that the P-loop residues Phe468 and Val471 were in the closest contact with the DFG motif of the activation segment, but did also interact with Lys601 and Leu597, respectively. The Phe468 P-loop residue was orientated towards the αC-helix, not the A-loop, in agreement with recently obtained crystal structures [9]. The average distance between the Cα atom of Phe468 and the Cα atom of Val600 was 7.7 Å. The calculated average total interaction energy between Phe468 and Val600 was only -0.64 ± 0.1 kcal/mol. However, as both these residues contain non-polar side chains, they could affect the conformational stability of the A-loop through hydrophobic interactions as has previously been suggested [4].

The overall results from this analysis indicate that the conformational stability of the A-loop in B-RAF^WT ^can mainly be explained by the strong electrostatic interactions between the residues joined in the HBN and in particular those between residues in the A-loop and the αC-helix.

### Molecular dynamics of B-RAF^V600E^

**Figure 3:**
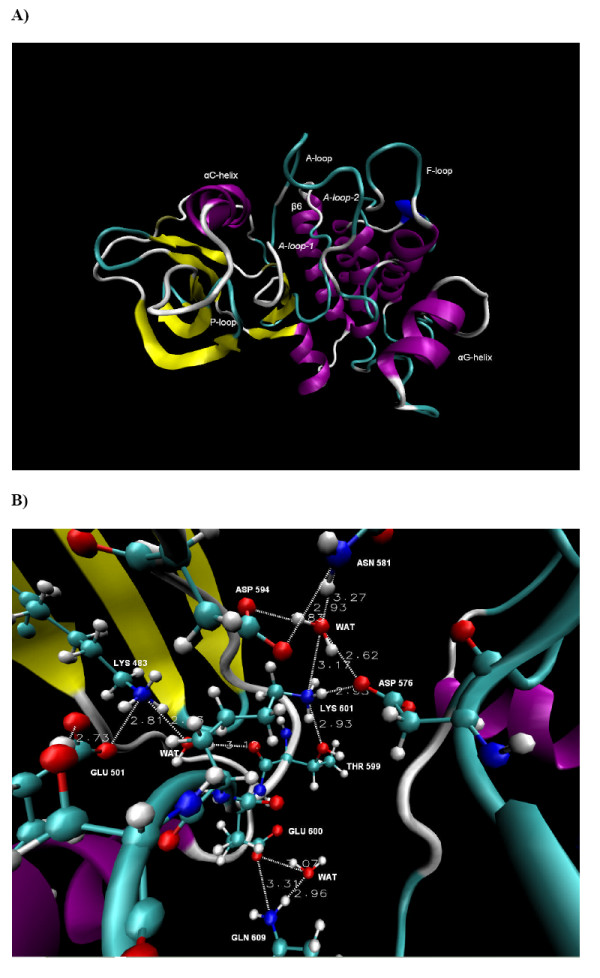
**B-RAF^V600E^**. **A) **The conformation of B-RAF^V600E ^after 15 ns of simulation time. The part of the A-loop resolved in the crystal structure (A-loop1) and the modelled portion (A-loop2) are shown. **B) **A close look at the conformation of the major catalytic residues and the identified hydrogen bond network in the unbounded B-RAF^V600E ^after 15 ns of simulation time. The dotted lines and the adjacent values represent the H-bonds and their distances.

The most frequent and one of the strongest activating B-RAF mutations in melanoma is the substitution Val for Glu at position 600. Thus Val, that has a small non-polar side chain, is replaced with an amino acid with larger and a negatively charged polar side chain. Figures 3A and 3B show the B-RAF^V600E ^protein conformation after 15 ns of MD simulation and a close view of the identified HBN site, respectively. During the simulation time significant changes were observed in the activation segment folding and in the position of other protein fragments compared with the B-RAF^WT ^(see Figures 2A and 3A). The A-loop adopted an open conformation and moved toward the space between αC-helix and β6-strand, indicating that the kinase underwent transition to the active form. The distance between the OG atom of Ser602 in the A-loop and the CD2 atom of Leu505 in the αC-helix decreased from 9.4 Å to 4.6 Å, indicating that the activation segment occupied the inhibitors binding site [4,9,11,12]. It should be mentioned, that the initial conformations for the MD simulations were taken from the co-crystallized protein-ligand complexes for both the B-RAF forms, and thus structural changes were expected during the first part of the simulations of the unbounded proteins.

The HBN observed in B-RAF^WT ^underwent significant changes in B-RAF^V600E ^(see Figures 2B and 3B). The water mediated hydrogen bond network HBN2, comprised of Asn581, Asp576, Asp594, Thr599 and Lys601, was disordered compared to the corresponding HBN2 in the wild type. The HBN1 portion, including the Lys483, Glu501 and Ser602, was not linked to HBN2 due to the disruption of the Lys483-Asp594 and Glu501-Lys601 ion pair's links. The average electrostatic interaction energy between Lys483 and Asp594 was almost two times less negative than in the wild type. Thus one of the important factors for stabilizing the A-loop conformation according to the results in the previous paragraph was not present in the B-RAF^V600E ^mutant.

Previously, a detailed analysis was performed of the structural changes within the HBN caused by the Val for Glu substitution at position 600 on the basis of a 10 ns simulation [11]. According to our hypothesis, the electrostatic interaction energy between Asp594-Lys601 and Asn581-Asp576 ion pairs becomes less negative at 4.5 ns due to conformational change of Lys601, which contributes to the Glu600 conformational change at 4.8 ns, causing the Glu600-αC-helix interaction energy to become less negative as well (see Figure Eight in [11]). Thus by analysing the evolution in the electrostatic energies of key substructures, we were able to identify some of the underlying processes that contribute to the overall destabilization of the A-loop conformation.

In order to strengthen the scientific basis for our hypothesis and obtain a more precise description, we made a similar study based on the 15 ns MD simulation presented in this paper. Thus curves for the electrostatic interaction energies corresponding to those previously published were made (not shown here). It was confirmed that the systems had already reached to an equilibrium in the 10 ns simulation, and maintained this equilibrium in the 10–15 ns interval, i.e. all the curves had a constant value in this interval.

The comparison with the B-RAF^WT ^results shows that the conformation of Lys601 and the layout of the HBN were affected by the Val to Glu600 residue replacement. The total interaction energy between the Asp594-Lys601 and Lys483-Glu501 ion pairs was 67.9 kcal/mol less negative than in B-RAF^WT ^due to the disruption of the H-bonds that linked these two ion pairs in the wild type B-RAF (see Table 1A). Thus our results indicate that the V600E substitution leads to more unfavourable A-loop-αC-helix interactions compared to the wild type, which can cause displacement of the A-loop. Conversely, these interactions were highly favourable in the wild type, stabilizing the A-loop kinase conformation (see Table 1A). The effect of the modelled portion of the A-loop was discussed previously, and based on the analysis of the electrostatic energy mentioned above, it was argued that the conformational changes of key residues like Glu600 and Lys601 were not significantly affected by this model [11].

**Figure 4:**
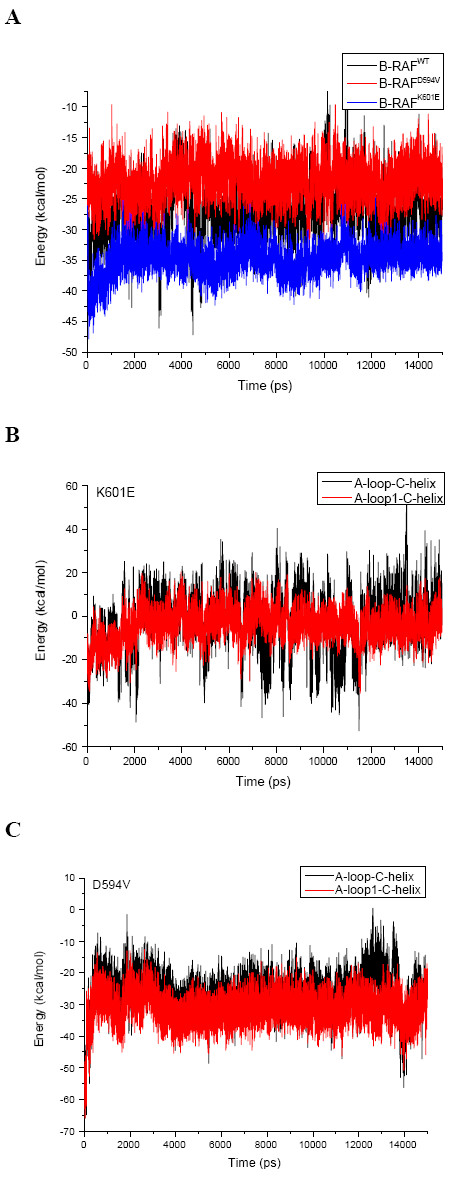
**Electrostatic energy interactions in B-RAF^WT^, B-RAF^D594V ^and B-RAF^K601E^**. **A) **The total electrostatic interaction energies between the A-loop and the P-loop in B-RAF^WT^, B-RAF^D594V ^and B-RAF^K601E^. **B) **The electrostatic interaction energy between the A-loop and the αC-helix, and the A-loop-1 (residues 593–602, the A-loop portion solved in the crystal structures) and the αC-helix in B-RAF^K601E^. **C) **The electrostatic interaction energy between the A-loop and the αC-helix, and the A-loop-1 and the αC-helix in B-RAF^D594V^.

Our results also showed that the A-loop-P-loop interactions were stable through the simulation and had similar total interaction energies in both the B-RAF^WT ^and B-RAF^V600E ^(see Table 1A and Figure 4A, as well as Figure Eight C in [11]). This indicated that the P-loop is unlikely to have a major contribution to the destabilization of the HBN in B-RAF^V600E^.

These results quantitatively demonstrate that the repulsive A-loop-αC-helix interactions and the interactions between the other participants in the HBN are likely to significantly affect the mechanism of the B-RAF^V600E^activation. The repulsive electrostatic forces between the A-loop, the αC-helix and the P-loop were identified as the main mechanism of B-RAF kinase activation in a previous MD study of the V600E and V599Ins mutants, but the specific contributions of the αC-helix and the P-loop were not specified [10].

The data above indicate the important role of the electrostatic interactions between the participants in the HBN in provoking different conformational changes in the different kinase types.

### Molecular dynamics of B-RAF^K601E^

The strong kinase activation in the presence of K601E mutations is difficult to explain in the frame of the previously published structural model in which mainly the interactions between the A-loop and the P-loops were considered to be of a key importance [4]. According to the B-RAF^WT ^MD results above and available crystal structures, the Lys601 is placed in front of and interacts strongly with the catalytic Asp594 residue. Our MD data for the wild type protein showed that this residue also interacts with the ATP coordinating residues Glu501 and Lys483. Thus, it is reasonable to suggest that mutation and subsequent conformational changes at this protein site would also promote significant changes in kinase activity. As Lys, an amino acid with a positively charged side chain, it is replaced with Glu that has a negatively charged side chain, the observed HBN is likely to be affected by this mutation.

**Figure 5:**
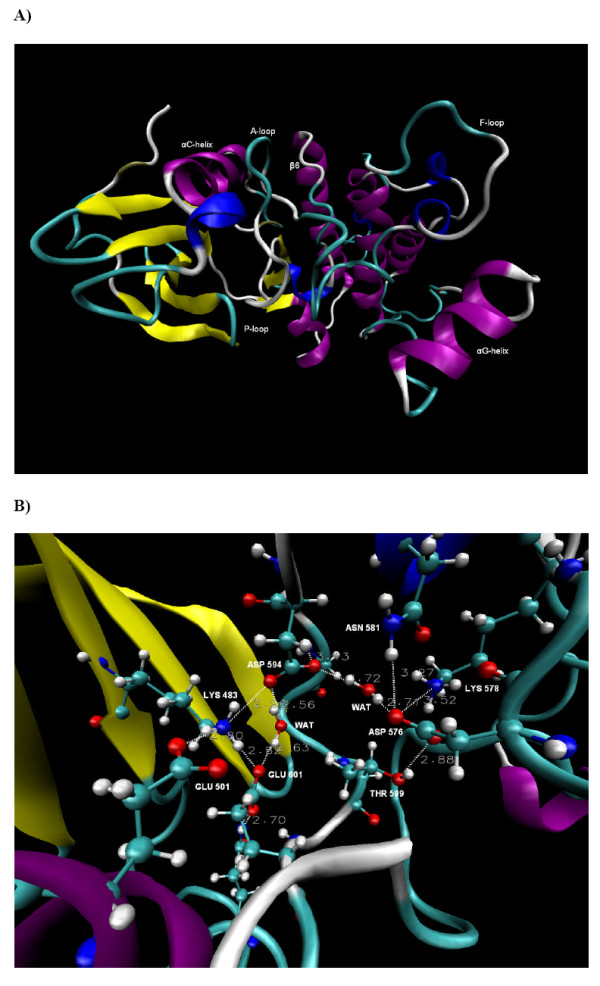
**B-RAF^K601E^**. **A) **The conformation of B-RAF^K601E ^after 15 ns of simulation **B) **A close look at the conformation of the major catalytic residues and the identified hydrogen bond network in the unbounded B-RAF^K601E ^after 15 ns of simulation time. The dotted lines and the adjacent values represent the H-bonds and their distances.

Figures 5A and 5B show the B-RAF^K601E ^protein conformation after 15 ns of MD simulation and a close view of the identified HBN site, respectively. The HBN observed in B-RAF^WT ^underwent significant changes compared to the wild type B-RAF, particularly due to disordering of the HBN1 portion that is comprised of Lys483, Glu501, Asp594, Glu601 and water molecules. In addition to forming H-bonds with Glu501 and Asp594, Lys483 is also hydrogen bonded to Glu601. All these H-bonds were stable during the whole simulation time, provoking conformational changes in HBN1 (see Figure 5B). It is notable that the Lys483-Asp594 had an average distance of 3.32 Å ± 0.96 and an H-bond was observed during 80% of the simulation time. However, the electrostatic interaction energy between these residues was less negative than in B-RAF^WT ^and at the same time more negative than in B-RAF^V600E ^(see Tables 1A and 1B). Thus, the Lys483-Asp594 link, identified as an important factor for the B-RAF^WT ^A-loop conformational stability in the analysis above, was shown to be weakened as a result of the Lys to Glu replacement at position 601, although to a lesser degree than for the V600E mutant.

The unfavourable interactions were mainly seen to be due to the Glu601-αC-helix repulsive interactions, in particular those between the Glu501 and the Glu601 residues. The Glu601-αC-helix electrostatic interaction energy was thus 62.5 kcal/mol less negative than the corresponding value for the Lys601-αC-helix interaction in B-RAF^WT ^and 12.0 kcal/mol less negative than in B-RAF^V600E^. The average electrostatic interaction energy between the Lys483-Glu501 and the Asp594-Glu601 ion pair's was 21.9 kcal/mol less negative than for the corresponding interaction in the wild type B-RAF. The Asp594-Glu601 electrostatic interaction had a positive value due to the repulsive forces between these two negatively charged residues, opposite to the corresponding strong attractive forces between Asp594 and Lys601 in both B-RAF^WT ^and B-RAF^V600E ^(see Tables 1A and 1B).

Similarly to the V600E mutant, our results indicate that the disordering of the HBN and the reduction of the electrostatic interaction energy between the A-loop and the αC-helix compared to the wild type is an important factor for B-RAF^K601E ^activation. Presumably, the introduction of stronger repulsive forces between these two protein substructures by this mutation, is one of the major factors contributing to the change of the A-loop to its active form. On the other hand, as the corresponding A-loop – P-loop energy was seen not to be affected by this mutation (see Table 1B), but P-loop residues might affect the A-loop conformation through hydrophobic interactions as previously discussed.

According to our results, the significant energy contribution came mostly from the residues in the A-loop-1 portion and the αC-helix (see Figure 4B), whereas the higher energy fluctuations were provoked by the modelled A-loop-2 portion. The average electrostatic energy between the A-loop and the αC-helix and the corresponding energy for A-loop1 reached a reasonable equilibrium after 2 ns of simulation time. As a result, the average electrostatic interactions energy between the A-loop and the αC-helix was -9.2 ± 12.2 kcal/mol, i.e. about 3–4 times less negative than in the wild type kinase (see Table 1B).

Considering the data from the MD simulation of B-RAF^V600Ins ^mutant [10] that indicated the same activation mode, we suggest that this mechanism of B-RAF activation by displacement of the A-loop is common in the most of the activating mutants. For instance, the mutation of Gly596 to larger and charged residues, as for example Arg596, would be expected to provoke a similar disruption of the electrostatic interactions between the A-loop and the αC-helix. These data demonstrate quantitatively that the changes in the HBN and the reorganisation of the catalytic and ATP coordinating residues joined in the HBN can contribute to the explanation of changes in the kinase activity and conformational stability.

### Molecular dynamics of B-RAF^D594V^

**Figure 6:**
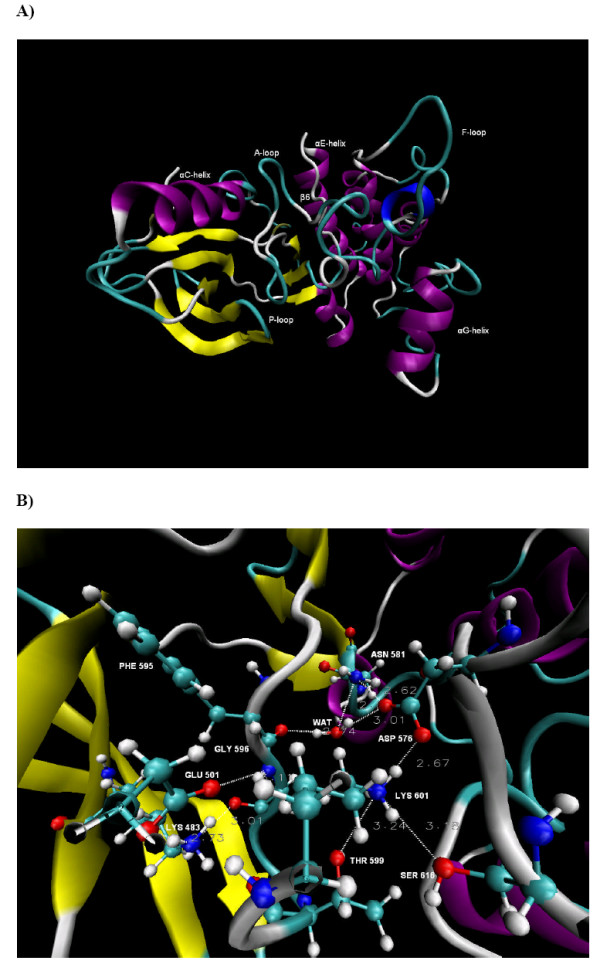
**B-RAF^D594V^**. **A) **The conformation of B-RAF^D594V ^after 15 ns of simulation time. **B) **A close look at the conformation of the major catalytic residues and the identified hydrogen bond network in the unbounded B-RAF^D594V ^after 15 ns of simulation time. The dotted lines and the adjacent values represent the H-bonds and their distances.

The Asp to Val substitution at position 594 eliminates the kinase activity, which would be expected due to the catalytic functions of aspartic acid in that position seen in many other kinases. Asp which has a negatively charged polar side chain is thus replaced with Val that has a non-polar side chain. Figures 6A and 6B show the B-RAF^D594V ^protein conformation after 15 ns of MD simulation and a close view of the identified HBN site, respectively. The most significant structural changes compared with B-RAF^WT ^were seen in the A-loop, the αE-helix and the F-loop. Similarly to the other mutants the HBN in B-RAF^D594V ^was disordered due to the D594V mutation. This substitution did lead to a change in the Phe595 conformation which adopted position that occupied the ATP binding site in a similar way as the inhibitors [4,9,11,12], restricting the flexibility of the A-loop.

The overall electrostatic interactions between the αC-helix and the A-loop were similar to the corresponding interactions in the wild type and much stronger than for the active mutants, B-RAF^V600E ^and B-RAF^K601E^, (see Figure 4C and Tables 1A–B). While the H-bonds involving Asp594 were disrupted due to this mutation, a strong average electrostatic interaction emerged between the Val594-Lys601 and the Asp576-Asn581 ion pair's. The Asp576 is within the β6-strand, indicating the A-loop – β6-strand interactions also contribute to the stability of this impaired activity mutant.

In the B-RAF^D594V ^mutant, as in the other studied kinases types, the portion of the A-loop solved in the crystal structures played a major role for the stability of these interactions. After 3 ns of simulation time the αC-helix-A-loop-1 electrostatic interactions reached a reasonable equilibrium at about -30 kcal/mol (see Figure 4C). The A-loop-2 portion had insignificant energy contribution with small fluctuations in the range of -5 to + 5 kcal/mol.

Our results indicate that this mutation thus stabilized the A-loop conformation and resulted in an auto-inhibition mechanism, most likely by occupying the ATP binding site by Phe595.

### Proposal for a molecular basis of the B-RAF activation

Based on the MD results above and all available crystal structures we propose a structural basis that could explain the experimentally observed B-RAF deviations in the kinase activity induced by several mutations. According to our hypothesis, the electrostatic interactions between the participants in the identified HBN in B-RAF^WT ^presumably play a major role in the kinase activity variations. Most of the residues joined in this network are either fractions of the A-loop or they are well known catalytic residues. Thus, the direct or indirect disordering of the HBN can lead to large kinase activity deviations and to conformational changes in the A-loop. Some mutations destabilize the HBN in such a way that it presumably promotes the kinase to change to its active form, while other mutations stabilize the A-loops conformation, causing the kinase to remain inactive.

It should be mentioned that very substantial structural changes provoked by the different mutations were observed in this work. Although some errors might be introduced through the MD simulation, the general trends seen in these simulations are very clear, and thus we consider our conclusions to be trustworthy.

The mutation of the residues that are joined in the identified B-RAF^WT ^HBN or are placed in neighbouring positions, are considered to directly disorder the HBN and as a consequence to affect the activation segments conformation. It has been established that Lys483 and Asp594 are highly conserved residues and of key importance for the ATP binding and coordination in the kinase family. Moreover, the Lys483-Glu501 ion pair interactions increase the phosphorylation potential [24,25]. According to our MD results, the substitution of Lys601 destabilized the interactions in the HBN, particularly affecting the interactions between the A-loop and the αC-helix and promoting significant conformational changes in the A-loop. Conversely, the Asp594 mutation rearranged the interactions in the HBN in such a way that strong A-loop – αC-helix interactions were maintained. The experimental results showed that B-RAF^K483M ^and B-RAF^D594V ^are the only known mutants with a lack of kinase activity, whereas the B-RAF^K601E ^is one of the four mutation positions that cause the strongest activation [4]. B-RAF^V600E ^is one of the most strongly activating [4] mutants. Based on our MD data for the V600E mutation demonstrated that Val for Glu substitution at position 600 (neighbour to the HBN participant Lys601) we observed both disordering of the HBN and increased repulsive electrostatic interactions between the A-loop and the αC-helix. Thus by replacing Val with a larger and more charged residue like Glu, increased repulsive forces between key residues were introduced, which according to our observations leads to breaking of the H-bonds between the αC-helix residue Glu501 and the A-loop residues Asp594 and Lys601. At the same time the activation segment is seen to move to a more open conformation, and it is our hypothesis that it moves to its active form. Other substitution of Val600 to large and charged residues, such as V600D, V600K and V600R, will most likely have similar impact on the Lys601 conformation and are therefore also expected to disorder the HBN and activate the B-RAF.

Several other residues with known mutations that affect the kinase activity are joined in the identified HBN or are placed at neighbouring positions and can thus directly destabilize the HBN and affect the A-loops position. It has been shown that the mutations of the Asn581, Thr599, Phe595 and Leu597 residues lead to significant increase in the kinase activity [4], whereas G596R mutant causes reduced activity compared to the B-RAF^WT ^basal activity (see Table S 1 in the supporting information of Ref [4]).

**Figure 7:**
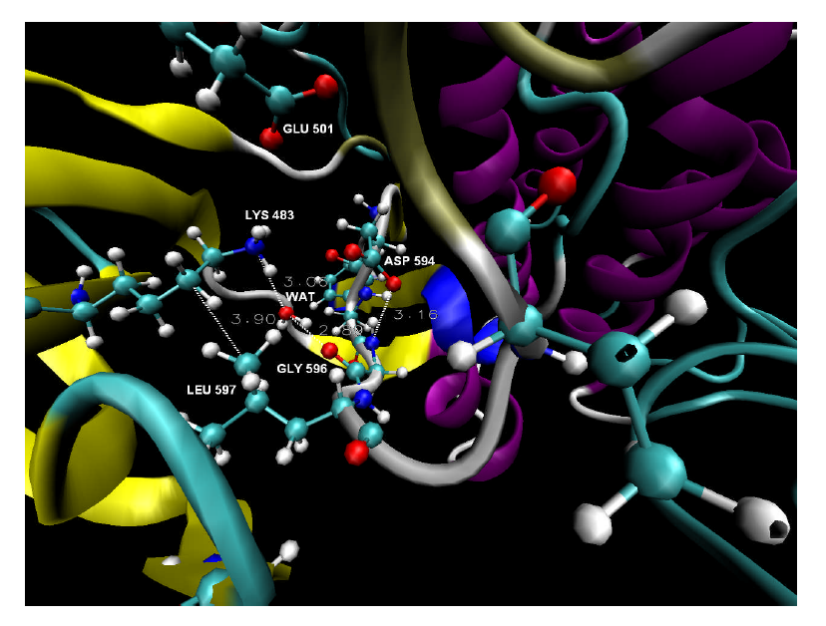
**Some of the residues participating in indirect disordering of the HBN in B-RAF^WT^**. A close look at the conformation of the major catalytic residues, the DFG motif and the P-loop residues participating in the disordering of the identified hydrogen bond network in the unbounded B-RAF^WT ^after 15 ns of simulation time. The dotted lines and the adjacent values present the H-bonds and their distances.

The Asn581 is a part of HBN2 by forming water mediated H-bond with Asp594 and a strong H-bond with the backbone nitrogen of Phe595 (see Figure 2A). Likewise, Thr599 forms a H-bond with Asp576 (average distance of 3.02 Å ± 0.39) which remained stable during 83% of the simulation time (see Figure 2A), thus the T599I mutation should induce changes in the HBN. Moreover, the phosphorylation of Thr599 will most probably destabilize the HBN and affect the conformation of the A-loop, which can explain why this process is needed for B-RAF^WT ^activation [5]. The substitution of the Phe595 residue (adjacent to the HBN participant Asp594) could potentially destabilize the HBN by flipping the conformation of aspartic acid. We observed that Leu597 makes a close contact and has strong van der Waals interactions with Lys483 (see Figure 7). Thus, the substitution of Leu597 (L597R) can presumably destabilize the interaction with Lys483 and consequently disrupt the HBN. Indeed such mutation will also disturb the hydrophobic interactions with the P-loop, as has been proposed previously [4]. It is reasonable to expect that the substitution of residues that disorder the HBN can affect the A-loop conformation. Further, the deactivating mutation of Gly596 (G596R) that is involved in the HBN1 portion of the network could form stable hydrogen bond with the catalytic Asp594 residue and water mediated H-bond with the ATP coordinating Lys483 residue (see Figure 7) and provoke rearrangements of the HBN.

The data from the MD simulations in a combination with the experimental results support our hypothesis and suggest that the mutations of residues in the identified HBN and in particular those which are in close contact with the catalytic residues are of a key importance for the kinase activity. It should be noted that mutations of residues like Val600, Lys601, Phe595, Gly596 and Leu597, which interact directly with the catalytic and ATP coordinating residues Lys483 and Asp594, promote stronger increase in kinase activity than residues like Thr599 and Asn581, which are a part of the HBN, but do not interact directly with the catalytic residues. (See Table S Four in the supporting information of ref [4]). This observation suggests an additional support and a logical explanation of the proposed model for explaining the experimentally observed activities of the mutants.

Mutations of residues that interact with the residues involved in the HBN can indirectly destabilise the HBN and consequently cause activity deviations. One of the strongly activating B-RAF mutations is G467A. As it has been previously suggested, this mutation could destabilize the interactions between the P-loop and the DFG motif due to the close contact with Leu597 seen in the crystal structures with the inhibitor Sorafenib [4]. However, our MD results suggest that Leu597 also makes a close contact with Lys483 and Gly467 and interacts with Met482 as well (see Figure 7). Thus, the mutation of the Gly467 could have indirect impact on the HBN. Several other mutations in the P-loop may affect the HBN stability in an indirect way and consequently affect the A-loop conformation too. Hence we propose a multiple mechanism of the B-RAF activation and conformational stability.

## Conclusion

A novel molecular basis of B-RAF activation was suggested that could explain the experimentally observed deviations in the kinase activity induced by several mutations. According to our analysis, the mutations which directly or indirectly destabilized the interactions between the residues within the identified hydrogen bond network in B-RAF^WT ^contribute to changes in the electrostatic interactions between the A-loop and the αC-helix. This mechanism presumably plays a major role in the B-RAF activation by promoting the activation segment to flip into an active form in the activating mutants. Conversely, in the mutants with an impaired kinase activity, such as B-RAF^D594V^, and in B-RAF^WT^, the A-loop and the αC-helix interactions stabilized the kinase inactive form according to our analysis. By analysing interactions and conformational changes involving the main known catalytic residues within B-RAF we have indentified significant structural changes that can to reasonable extent explain activity deviations caused by several mutations.

## Authors' contributions

FFF performed the calculations. Both authors jointly analysed the results and wrote the article together.
